# Impact of the Norwegian Agreement for a More Inclusive Working Life on diagnosis-specific sickness absence in young adults: a difference-in-difference analysis

**DOI:** 10.1186/s12889-022-12636-9

**Published:** 2022-02-04

**Authors:** Rachel L. Hasting, Suzanne L. Merkus, Therese N. Hanvold, Petter Kristensen, Jon Michael Gran, Ingrid S. Mehlum

**Affiliations:** 1 Department of Occupational Medicine and Epidemiology, National Institute of Occupational Health, PB 5330 Majorstuen, 0304 Oslo, Norway; 2 Department of Occupational Health Surveillance, National Institute of Occupational Health, Oslo, Norway; 3 Oslo Centre for Biostatistics and Epidemiology, Department of Biostatistics, University of Oslo, Oslo, Norway; 4 Oslo Centre for Biostatistics and Epidemiology, Oslo University Hospital, Oslo, Norway; 5 Department of Community Medicine and Global Health, Institute of Health and Society, University of Oslo, Oslo, Norway

**Keywords:** Cohort study, Difference-in-difference, Gender, Mental health, Musculoskeletal diagnosis, Musculoskeletal disorder, Policy interventions, Psychological diagnosis, Register-based study, Sick leave

## Abstract

**Background:**

The Norwegian Agreement for a More Inclusive Working Life (the IA Agreement) aims to reduce sickness absence (SA) and increase work participation. Potential impacts of the IA Agreement have not been thoroughly evaluated. The study aimed to estimate the impact of the IA Agreement on musculoskeletal and psychological SA prevalence and duration among young adult men and women, and to identify whether the impact was modified by economic activity or SA grade.

**Methods:**

Data from national registries were combined for 372,199 individuals born in Norway 1967–1976. ICPC-2 codes identified musculoskeletal (L) and psychological (P) diagnoses. A difference-in-difference method compared prevalence and mean duration of first SA > 16 days between 2000 and 2005 separately for men and women working in IA companies relative to non-IA companies. Analyses were adjusted for mean company size and stratified by economic activity and SA grade (full/graded). Average marginal change was calculated with 95% confidence intervals (CI).

**Results:**

The impacts of the IA Agreement on SA prevalence were mixed as the direction and size of marginal changes varied according to diagnosis, gender, and economic activity. However, there was a general tendency towards reduced mean SA duration for both diagnosis groups, and in particular men with musculoskeletal SA (− 16.6 days, 95% CI -25.3, − 7.9). Individuals with full SA in IA companies had greater reductions in mean SA duration. Only the wholesale and retail economic activity indicated a beneficial contribution of the IA Agreement for both SA prevalence and duration, in both diagnoses and genders.

**Conclusions:**

Potential impacts of the IA Agreement on SA in young men and women varied according to diagnosis and economic activity. However, results indicated that the IA Agreement could reduce SA duration. Further research should identify reasons for gender and economic activity differences.

**Supplementary Information:**

The online version contains supplementary material available at 10.1186/s12889-022-12636-9.

## Background

Absence from work due to illness has a financial and social impact on multiple levels of society. For individuals, frequent and/or long sickness absence (SA) episodes can contribute to an inability to continue in employment [1], a loss of social interaction with colleagues, and a lower income. Nationally, high costs and productivity losses are associated with SA; in 2018, European Union states combined spent 1% (roughly €160 billion) of their GDP on sickness benefits [2]. These challenges have resulted in an increased focus on measures to prevent and reduce SA in the working population.

**Fig. 1:**
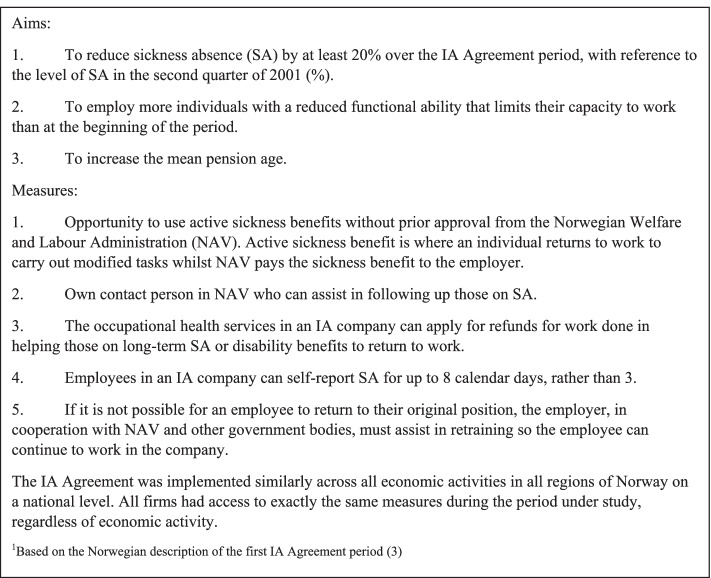
Characteristics of the first IA Agreement period, 2001–2004^1^

In Norway, an increasing number of individuals received SA and other benefits during the latter half of the 1990s [3]. Due to this, the Agreement for a More Inclusive Working Life (the IA Agreement) was developed in 2001 by the Government and organisations representing employers and employees. The aim was to, over a period of 4 years, reduce SA by 20% from the 2001 level and increase work participation [3]. Companies who signed the IA Agreement (so-called IA companies) gained access to different resources, including measures to prevent SA (e.g. workplace risk assessment training) and to aid in faster return to work (e.g. grants to help modify the workplace) [3]. See Fig. 1 for more details. Though not an IA Agreement measure, utilising graded SA instead of full SA has also been a main focus; it involves working part-time whilst on SA, and thus aids faster return to work [4]. In 2018, around 30% of companies were IA companies, covering almost 60% of the Norwegian working population [5]. The IA Agreement is now in its fifth term (2019–2022), where it has been extended to include all Norwegian companies [6].

During the IA Agreement period 2001–2018, the percentage of available working time lost to SA in the Norwegian working population dropped from 6.6 to 5.8%, a relative reduction of 12.4% [4]. To what degree this reduction can be explained by implementation of the IA Agreement, or measures such as graded SA, remains unclear. Few peer-reviewed studies have evaluated it, and results have been mixed [7–9]. Furthermore, the reduction in SA varied according to gender and economic activity. During the first 10 years of the IA Agreement, women’s SA decreased to a smaller extent than men’s, widening the existing gender gap [5]. SA was also reduced in some economic activities more than in others from 2001 to 2018, from a 36% reduction in the hotels and restaurants economic activity to less than 10% in education [4]. Recent studies have indicated the IA Agreement may play a role in these variations, and that any impacts may also differ between genders within an economic activity [7, 9, 10]. As the most recent version of the IA Agreement includes an economic activity-specific focus [11], understanding these differences will aid implementation and future evaluation of the IA Agreement. It could also provide valuable information for other countries looking to implement national interventions to reduce SA.

The IA Agreement’s effect on SA in young adults is of particular importance, as experiencing SA early in the working career can contribute to increased SA and lower income later in life [12]. Younger adults also show a larger gender gap in SA than other ages (e.g. over 3 percentage points (PP) in those aged 30–34, compared to 2 PP in those aged 45–49) [13]. This is partially, but not wholly, explained by pregnancy-related SA [13, 14]. Exploring the impact of the IA Agreement on younger adults will thus provide further insight into how the related measures work in this vital population.

The most common cause of SA in Europe is musculoskeletal disorders [15], which, along with psychological diagnoses, are receiving an increasing level of attention due to their consequences for individual wellbeing, work productivity, and costs [16]. These are also the two largest diagnosis groups in Norway, accounting for over 50% of SA in 2019 [17]. They respond differently to workplace interventions [18]; accordingly, grants and measures included in the IA Agreement will likely be utilised differently depending on the diagnosis. Thus, it is beneficial to study the diagnoses groups separately.

This paper aimed to estimate whether the IA Agreement had an impact on musculoskeletal and psychological SA in young adults. Two main research questions were addressed: 1) What is the impact of the IA Agreement on the prevalence and duration of musculoskeletal and psychological SA separately for men and women, and 2) To what extent is any impact modified by economic activity and grade of SA (full/graded)?

## Methods

### Data sources

The project group established a birth cohort in 2002 that is comprised of all individuals live-born in Norway in the period 1967–1976 (*n* = 626,928) [19]. Data from this cohort was used by linking different registries using the unique individual identification number. Information on gender, SA and economic activity were obtained from “FD-Trygd”, an events database on employment and welfare maintained by Statistics Norway (SSB) [20]. Data on company size (number of employees) were obtained from the Central Register of Establishments and Enterprises, also maintained by SSB [21]. Data on if/when companies entered into the IA Agreement, any changes to their agreement status, and SA diagnoses were obtained from the Norwegian Labour and Welfare Administration (NAV). Ethical approval was obtained from the South-East A Regional Committee for Medical and Health Research Ethics (case number 17344).

### Study design

A difference-in-difference (DID) method was used. We compared individuals working in companies with the IA Agreement (intervention) with those without the IA Agreement (controls), before implementation (2000) and after the first IA term (2005).

The DID method uses observational data to infer effects of quasi-experiments by comparing the outcome variable of the intervention group with a control group. A key assumption is that the intervention and control groups would have had the same trend over time for the outcome if there had been no intervention (the “common trends” assumption) [22]. This is usually tested by comparing the trend in outcome prior to the study period for each group. In the years prior to 2000, only a few of our young and healthy study population were diagnosed with musculoskeletal or psychological disorders. Therefore, we checked the trends in SA regardless of diagnosis (see Supplementary Fig. 1 and 2). The change over time for the intervention and control groups, in men and women respectively, appeared to be similar. Another key assumption is that the allocation of the intervention does not depend on the outcome pre-intervention [22]. The outcome in this study was SA at an individual level, and there is little to suggest that individuals select companies/jobs based on IA status [23]. IA was also allocated at a company level. Therefore, we consider this assumption to be reasonable. DID does not require that the individuals in each group are the same over time, but that the group characteristics are the same [24]. We checked the composition of the intervention and control groups respectively with regards to gender composition, age, company size, and economic activity, and they appear to be similar in 2000 and 2005 (Table 1). If these assumptions are fulfilled, DID can be used as an alternative where a randomised controlled trial (RCT) is not possible, allowing for the discussion of intervention effects [22].

**Table 1:** Descriptive statistics for employees in the intervention group (IA) and control group (non-IA) in 2000 and 2005 (*N* = 372,199)

	Intervention						Control					
	2000 *N* = 39,762 (20%)			2005 *N* = 71,699 (27%)			2000 *N* = 156,464 (80%)			2005 *N* = 191,731 (73%)		
	*N*	*%*	*Quartiles*	*N*	*%*	*Quartiles*	*N*	*%*	*Quartiles*	*N*	*%*	*Quartiles*
Year of birth			1969–1971-1976			1969–1971-1976			1969–1972-1976			1969–1971-1976
Female	25,265	64		40,390	56		68,137	44		73,584	38	
Company size			45–135-549			42–121-521			8–24-100			8–21-69
Economic activity												
*Agriculture/forestry/fishing*	97	< 1		130	< 1		4012	3		2802	1	
*Mining/quarrying*	508	1		1254	2		1562	1		4437	2	
*Manufacturing*	5066	13		11,041	15		20,072	13		25,474	13	
*Electricity/gas/water supply*	196	< 1		460	1		719	< 1		1008	1	
*Construction*	1276	3		2920	4		12,022	8		18,283	10	
*Wholesale/retail*	1442	4		2882	4		36,216	23		46,615	24	
*Hotels/restaurants*	695	2		585	1		9850	6		5196	3	
*Transport/storage*	2195	6		3504	5		14,510	9		15,276	8	
*Financial/real estate*	1901	5		4107	6		27,218	17		35,824	19	
*Public administration*	3760	9		7723	11		7167	5		6266	3	
*Education*	5577	14		10,881	15		2886	2		5305	3	
*Health/social*	15,887	40		24,860	35		11,864	8		17,216	9	
*Other*	1162	3		1352	2		8366	5		8029	4	

The intervention effect is captured using an interaction term between group (intervention/control) and time period (before/after intervention) in a regression model. A positive estimate indicates an increase in the outcome due to the intervention (relative to controls), whilst a negative estimate indicates a decrease in the outcome due to the intervention.

### Study population

**Fig. 2:**
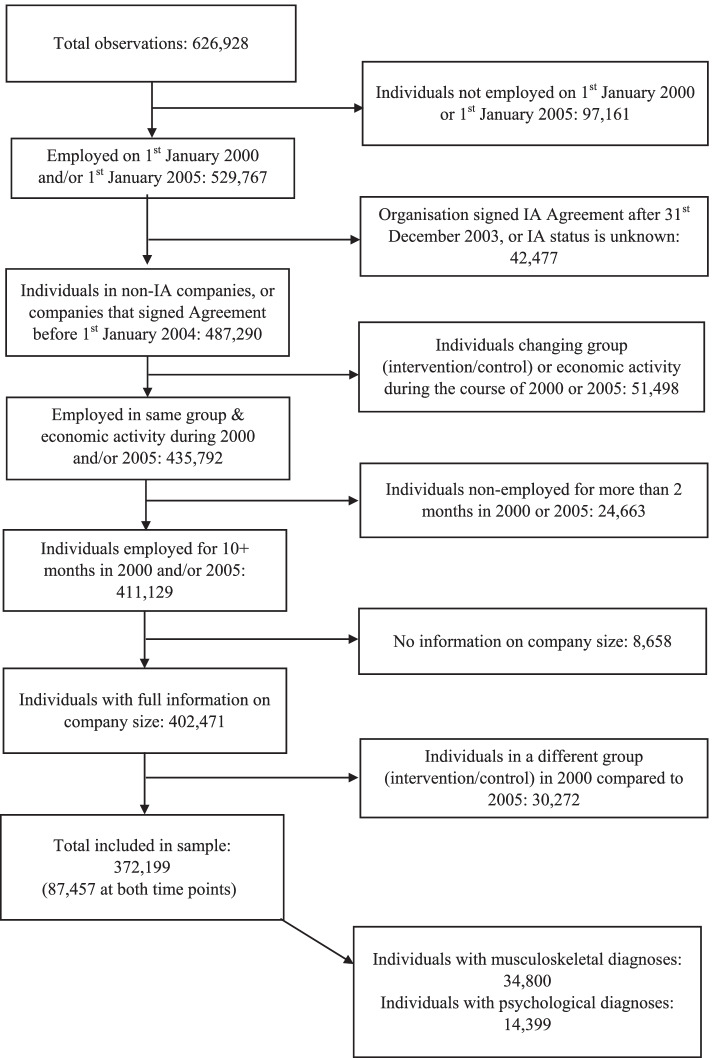
Flowchart of study population

The initial population consisted of 529,767 individuals, who were registered as employed in Norway on 1st January 2000 and/or 1st January 2005 (Fig. 2). Individuals were excluded in 2000 and/or 2005 if they worked for a company that signed the IA Agreement after 1st January 2004. This was to ensure that the intervention was implemented at approximately the same time. Individuals were also excluded if they or the company changed group (intervention/control) or economic activity during the course of 2000 or 2005, were non-employed for more than 2 months of the year, or had missing information on company size. Finally, for those present in both years, individuals were excluded if they were in a different group (intervention/control) in 2005 compared to 2000. In total, 157,568 individuals (30%) of the initial population were excluded.

### Study outcomes

In Norway, sickness benefits are covered by the employer for the first 16 calendar days of SA. After this, the responsibility is passed on to NAV and the entire episode is registered in the sickness benefits database. This information is passed on to SSB and incorporated in the FD-Trygd database, from which we obtained SA data. Thus, only absences > 16 days are included to ensure records are complete. Individuals must obtain a sickness certificate from their physician, with a diagnosis denoted by an International Classification of Primary Care (ICPC-2) code [25].

The two main study outcomes were one-year SA prevalence (at least one SA episode > 16 days) and the duration of the first SA episode for those with SA > 16 days during 2000 and 2005, respectively. The outcomes were studied separately for musculoskeletal diagnoses (ICPC-2 code L) and psychological diagnoses (ICPC-2 code P), and for men and women. We first analysed both full (100%) and graded (< 100%) SA together, before analysing them separately and comparing the results with our main analyses to identify whether the IA Agreement influenced the implementation of graded SA. For SA episodes that began in the previous year (i.e. 1999 or 2004) or continued further into the following year, the duration of the entire SA episode was used, including days beyond 2000 or 2005.

### Covariates

Covariates included in this study were gender, economic activity, and mean company size. The economic activity variable was coded according to the Standard Industrial Classification 2002 [26], based on the Statistical Classification of Economic Activities in the European Community (NACE) Revision 1.1, and included 13 different economic activity categories (see Table 1). Some economic activities were grouped together in categories in order to increase sample size for analyses; these were agriculture/forestry/fishing (categories A and B), financial/real estate (J and K), and other (O, P, and Q). As some individuals worked at multiple companies during the year, the mean company size was calculated for each individual.

### Statistical analyses

All analyses were carried out in Stata, version 15.1 [27].

Linear probability models were used to calculate the average marginal change in the one-year prevalence of SA (measured in percentage point (PP) change). Due to the skewed nature of the data, negative binomial regression models were used to calculate the average marginal change in the duration of first SA episode (measured in days). When the number of individuals in the stratified economic activities exceeded 500 both in 2000 and 2005, we ran economic activity-specific models. Full/graded SA analyses were carried out on the same economic activities, even when sample size < 500. The “margins” command in Stata was used to calculate average marginal change. 95% confidence intervals (CI) were calculated using clustered standard errors at the individual level, to account for correlation between individuals present at both time points.

## Results

The final study population comprised 372,199 individuals (70% of the initial employed population), of whom 87,457 were present at both time points (Fig. 2). For the purposes of studying SA duration, only those with SA episodes (all-cause) were included; this resulted in two subpopulations of 34,800 for musculoskeletal SA and 14,399 for psychological SA.

Table 1 shows the background characteristics for the intervention (IA) and control (non-IA) groups in the main population (see Supplementary Table 1 for the subpopulations). There was a higher proportion of individuals in the control group both in 2000 and 2005 (80 and 73%, respectively); women were more likely to be in the intervention group at both time points (64 and 56%, respectively). The median company size was well over 100 employees in both years for those in the intervention group, and below 25 employees for the control group. Individuals in IA companies were more likely to work in the health and social economic activity (40% in 2000; 35% in 2005) and education economic activity (14% in 2000; 15% in 2005), whilst individuals in non-IA companies were more likely to be in wholesale/retail (23% in 2000; 24% in 2005) and financial/real estate (17% in 2000; 19% in 2005) economic activities. For a more detailed breakdown of the distribution of gender and diagnoses within economic activities, see Supplementary Tables 4 and 5.

In our total sample in 2000, the prevalence of musculoskeletal SA was 7.5% for men and 10.0% for women, whilst for psychological SA the prevalence was 2.1 and 3.5%, respectively. The mean duration (days) of musculoskeletal SA in 2000 for men was 102 (standard deviation (SD) 101, interquartile range (IQR) 30–56-133) and for women was 106 (SD 95, IQR 36–71-140). For psychological SA, this was 125 (SD 115, IQR 34–74-189) days for men, and 124 (SD 115, IQR 34–73-176) days for women.

### Main analyses

#### Musculoskeletal diagnoses

Musculoskeletal SA prevalence was lower in 2005 than in 2000, in both genders and both groups (intervention/control) (Table 2). The negative DID estimate for women indicates that the decrease occurred to a larger extent in the intervention group than in the control group. For men, the positive DID estimate indicates that a decrease occurred to a lesser extent in the intervention group compared to the control group.

**Table 2:** Prevalence and duration of musculoskeletal and psychological SA for male and female employees in the intervention group (IA) and control group (non-IA) in 2000 and 2005, the change over time, and difference-in-difference (DID) estimates (N = 372,199)

	Intervention								Control									
	2000 *N* = 39,762 (20%)			2005 *N* = 71,699 (27%)			Change 2000–2005		2000 *N* = 156,464 (80%)			2005 *N* = 156,464 (80%)			Change 2000–2005		DID Estimate^a^	
	*N (%)*	*Mean (SD)*	*Median (IQR)*	*N (%)*	*Mean (SD)*	*Median (IQR)*	*PP*	*Days*^*b*^	*N (%)*	*Mean (SD)*	*Median (IQR)*	*N (%)*	*Mean (SD)*	*Median (IQR)*	*PP*	*Days*^*b*^	*Marginal Change*	*95% CI*
Musculoskeletal (code L)																		
*Prevalence*																		
*Men*	1033 (7.1)			2014 (6.4)			−0.7		6626 (7.5)			7895 (6.7)			−0.8		0.3	− 0.2, 0.9
*Women*	2543 (10.1)			3383 (8.4)			−1.7		6380 (9.4)			6061 (8.2)			−1.2		−0.4	− 0.9, 0.1
*Duration (days)*																		
*Men*		98 (102)	54 (28–128)		93 (100)	48 (27–115)		−5		102 (101)	57 (30–134)		115 (113)	62 (32–157)		13	**−16.6**	**−25.3, −7.9**
*Women*		98 (89)	67 (34–128)		113 (113)	62 (31–156)		15		109 (96)	73 (37–146)		129 (120)	74 (34–195)		20	−4.3	−10.9, 2.3
Psychological (code P)																		
*Prevalence*																		
*Men*	337 (2.3)			708 (2.3)			0.0		1801 (2.0)			2602 (2.2)			0.2		−0.1	−0.4, 0.3
*Women*	914 (3.6)			2184 (5.4)			1.8		2313 (3.4)			3741 (5.1)			1.7		0.2	−0.2, 0.6
*Duration (days)*																		
*Men*		114 (112)	59 (30–162)		108 (109)	58 (29–145)		−6		127 (116)	76 (34–193)		129 (121)	73 (33–199)		2	−6.1	−22.2, 10.0
*Women*		111 (109)	62 (31–157)		125 (118)	74 (32–190)		14		128 (117)	77 (35–190)		142 (125)	87 (36–240)		14	−1.4	−12.3, 9.5

^a^ Analyses adjusted for economic activity (13 categories) and mean company size. Bold font indicates estimates are statistically significant at the 5% level

^b^ Calculated using mean days

*SA* sickness absence, *IQR* interquartile range, *CI* confidence interval, *SD* standard deviation, *PP* percentage points

Median SA duration for musculoskeletal SA was lower in the intervention groups in 2005 compared to 2000, but higher in the control groups, for both men and women. The negative DID estimate for women indicates that the intervention group increased to a lesser extent than the control group. In men, the negative DID estimate reflects the decrease in SA duration in the intervention group relative to the increase in the control group (− 16.6 days, 95% CI -25.3, − 7.9; see Table 2).

Stratification on grade of SA showed larger negative DID estimates for full SA than for graded SA for prevalence in women and for duration in both genders (Table 3). The change in DID estimates was particularly prominent for SA duration in women, where the difference in full SA was almost 5 days larger compared to the original estimate. However, for prevalence in men, there was a slightly larger positive DID estimate for full SA, compared to the original estimate, and a weak negative estimate for graded SA.

**Table 3:** DID estimates for SA prevalence and duration in those with graded (< 100%) SA and those with full (100%) SA, compared to original analysis^a^

	Original Analysis		< 100% SA		100% SA	
	*Marginal Change*	*95% CI*	*Marginal Change*	*95% CI*	*Marginal Change*	*95% CI*
Musculoskeletal (code L)						
*Prevalence (PP)*						
*Men*	0.3	−0.2, 0.9	− 0.2	− 0.5, 0.1	0.5	− 0.0, 1.0
*Women*	− 0.4	− 0.9, 0.1	− 0.1	−0.4, 0.3	− 0.4	−0.8, 0.1
*Duration (days)*						
*Men*	**−16.6**	**−25.3, −7.9**	− 12.2	−32.8, 8.4	**− 17.7**	**− 27.3, −8.1**
*Women*	−4.3	− 10.9, 2.3	−2.7	− 13.9, 8.5	**−9.1**	**− 17.4, − 0.8**
Psychological (code P)						
*Prevalence (PP)*						
*Men*	−0.1	− 0.4, 0.3	0.0	− 0.1, 0.2	− 0.1	−0.4, 0.2
*Women*	0.2	−0.2, 0.6	0.2	−0.1, 0.4	0.1	−0.2, 0.4
*Duration (days)*						
*Men*	−6.1	−22.2, 10.0	22.3	−9.2, 53.9	−13.2	−31.7, 5.3
*Women*	−1.4	−12.3, 9.5	−3.1	−22.2, 16.1	−5.4	− 19.0, 8.2

^a^ Analyses adjusted for economic activity (13 categories) and mean company size. Bold font indicates estimates are statistically significant at the 5% level

*PP* percentage points, *CI* confidence interval

### Psychological diagnoses

For psychological SA, men in both groups had a lower median duration in 2005 compared to 2000, whilst women had a higher median duration in both groups (Table 2). The negative DID estimates indicate that the mean duration in men decreased more in the intervention group than in the control group, whilst for women, mean duration increased less in the intervention group than in the control group.

For both genders, the trends in prevalence persisted regardless of grade of SA (Table 3). For SA duration, both genders had larger negative DID estimates with full SA, compared to the original analysis. Men in the intervention group showed an increase in duration of graded SA, compared to the original analyses.

### Economic activity-specific analyses

#### Musculoskeletal diagnoses

The direction of the DID estimates for the prevalence of musculoskeletal diagnoses varied between the economic activities (Table 4). Regarding mean duration within the economic activities, the intervention group generally showed a larger decrease between 2000 and 2005 compared to the control group, especially in men. The DID estimates for prevalence and mean duration generally tended to have the same direction within economic activity for each gender, with some exceptions (e.g. men in the transport/storage, financial/real estate, and health/social economic activities). However, the estimates generally varied between genders within an economic activity. Only the wholesale/retail economic activity showed consistent negative DID estimates across outcomes and genders.

**Table 4:** Results of the difference-in-difference (DID) analyses for musculoskeletal- and psychological-related prevalence and duration, when stratifying by economic activity and gender^a^

	Musculoskeletal (code L)				Psychological (code P)			
	Prevalence (PP)		Duration (days)		Prevalence (PP)		Duration (days)	
	*Marginal Change*	*95% CI*	*Marginal Change*	*95% CI*	*Marginal Change*	*95% CI*	*Marginal Change*	*95% CI*
Manufacturing								
*Men*	−0.9	−2.3, 0.4	**−19.5**	**−34.1, −4.9**	−0.4	− 1.1, 0.3	−8.5	− 42.6, 25.6
*Women*	1.0	−1.3, 3.4	4.3	−18.8, 27.4	−0.4	− 1.8, 1.0	18.8	−25.0, 62.7
Construction								
*Men*	−1.4	−3.8, 1.1	−21.5	−44.2, 1.2	0.0	−1.0, 1.1	−32.2	−93.7, 29.3
Wholesale/retail								
*Men*	−0.3	−2.5, 1.8	−10.1	−44.7, 24.4	**−1.6**	**−3.1, − 0.1**	−11.0	−65.3, 43.4
*Women*	−2.7	−5.8, 0.3	−18.2	− 51.6, 15.1	− 0.9	−2.6, 0.7	−31.4	−94.1, 31.4
Transport/storage								
*Men*	1.2	−1.0, 3.4	−4.1	−29.5, 21.4	−0.5	−1.8, 0.8	**45.7**	**4.6, 86.7**
*Women*	1.3	−1.7, 4.3	22.7	−4.5, 49.9	0.9	−1.1, 2.9	0.3	−50.3, 50.8
Financial/real estate								
*Men*	0.5	−1.1, 2.0	−9.9	−65.2, 45.3	0.1	−0.9, 1.1	**55.6**	**4.8, 106.3**
*Women*	−1.4	−3.6, 0.9	−5.0	−34.0, 23.9	0.2	−1.4, 1.7	−30.5	−81.9, 20.8
Public administration								
*Men*	1.0	−0.4, 2.5	23.8	−10.1, 57.7	0.8	−0.1, 1.7	−9.0	− 75.6, 57.6
*Women*	−1.1	−3.1, 0.9	0.1	−30.5, 30.7	−0.5	−2.0, 0.9	1.7	−47.8, 51.2
Education								
*Men*	−0.1	−1.8, 1.5	−19.9	−69.7, 29.9	0.2	−1.2, 1.6	−38.6	− 102.6, 25.4
*Women*	0.4	−1.3, 2.0	−16.5	−44.2, 11.2	−0.1	− 1.5, 1.3	20.9	− 18.2, 60.0
Health/social								
*Men*	0.3	−1.4, 2.1	−26.4	−59.4, 6.6	1.2	−0.2, 2.5	−22.3	−70.8, 26.2
*Women*	−0.1	−1.1, 0.9	−4.5	−15.0, 6.0	0.1	−0.7, 0.8	− 7.1	−23.9, 9.7

^a^ Analyses adjusted for mean company size. Bold font indicates estimates are statistically significant at the 5% level. *PP* percentage points, *CI* confidence interval

For both genders, the DID estimates for the intervention group were generally larger for negative estimates and smaller for positive estimates when considering SA duration in full SA, compared to the original estimates. In contrast, estimates were smaller for negative estimates and larger for positive estimates when considering both SA prevalence and duration in graded SA, compared to the original estimates (Supplementary Table 2).

### Psychological diagnoses

There were no clear trends for the direction of economic activity-specific DID estimates for psychological diagnoses, neither within outcome nor for each respective gender (Table 4). Men in the intervention group showed a significantly larger increase in SA duration than the control group in the transport/storage (45.7 days, 95% CI 4.6, 86.7) and financial/real estate (55.6 days, 95% CI 4.8, 106.3) economic activities. Similar to the results for musculoskeletal diagnoses, in the wholesale and retail economic activities, the IA Agreement was consistently associated with a smaller increase in both outcomes in the intervention group compared to the control group. This was significant for SA prevalence in men (− 1.6 PP, 95% CI -3.1, − 0.2).

There were no clear trends in the direction of DID estimates for full or graded SA on psychological related SA prevalence or mean duration across economic activities (Supplementary Table 3).

## Discussion

This study used a difference-in-difference method to investigate the impact of the IA Agreement on the prevalence and mean duration of sickness absence separately for young men and women with musculoskeletal and psychological diagnoses, and to identify whether economic activity and graded SA modified these effects. Our results indicate that there are differences between those with and without the IA Agreement, as those working in companies with the IA Agreement tended to have a shorter mean duration of both musculoskeletal- and psychological-related SA. This result was even stronger when considering only those on full SA. The potential impact of the IA Agreement on men and women varied according to economic activity. The only clear trend in DID estimates was observed in the wholesale and retail economic activity, which showed consistent benefits for both prevalence and mean duration in both diagnoses and both genders for those working in companies with the IA Agreement.

Previous evaluations of the IA Agreement come from reports and peer-reviewed studies, and suggest either a positive effect [5, 8, 9, 23, 28], or no significant effect on overall SA [7, 10, 29]. Our results indicate a general beneficial contribution of the IA Agreement towards reduced duration of both musculoskeletal and psychological diagnoses in both genders, particularly in men, and a mixed contribution with regards to prevalence. However, few of these estimates were statistically significant, meaning that these trends could be due to chance.

If the trends can be attributed to the IA Agreement, our results could indicate that the measures included in the IA Agreement contribute more towards faster return to work than prevention of initial SA. This is supported by the fact that many of the IA-related measures focused on longer-term SA are related to maintaining contact with the individual and adjusting the workplace to ensure faster return to work [11]. Graded SA also has the same aim [4]. Full SA episodes were generally shorter than graded SA episodes for those with the IA Agreement compared to controls. This could indicate that IA companies facilitate for graded SA to ensure the individual can participate in working life, where the individual would ordinarily have continued with full SA.

Economic activities varied in how and to what extent the IA Agreement impacted SA prevalence and duration. As mentioned, only the wholesale/retail economic activity showed a consistent beneficial impact of the IA Agreement on both prevalence and duration, though many economic activities showed a beneficial trend with regards to mean SA duration. Only musculoskeletal SA appeared to show a trend towards an impact of the IA Agreement on full and graded SA, with shorter SA episodes on full SA and more frequent, longer SA episodes with graded SA. Economic activity seems therefore to have a modifying effect on any potential impact of the IA Agreement on SA, which is in line with previous studies and reports [5, 7, 10]. Potential explanations for differences between economic activities may lie in how much effort economic activities have put into implementing the IA Agreement [7], or through the degree of manual labour involved and the ease with which tasks can be adjusted [10]. We did not have information relating to potential differences in the level of effort available in this study, but it is possible that economic activities do have differing levels of effort into implementation [30]. We did find that economic activities that tend to be associated with manual labour (e.g. wholesale/retail and construction) also tended to show a beneficial impact of the IA Agreement. However, we also found a similar result for psychological-related SA, which is not necessarily correlated with manual labour and has been shown to respond differently to workplace interventions [18]. We did not find any clear trends relating to gender within the economic activities, indicating that economic activity may play more of an important role than gender when considering the effectiveness of IA-related measures.

### Methodological considerations

This study used the DID method, which aims to observe and evaluate effects of quasi-experiments, such as the IA Agreement, where no large-scale RCTs are possible. However, DID includes assumptions that are very difficult to test in practice [22]. We were able to visually inspect the trend in all-cause SA in our study population prior to study start, but the young age of the study population meant we could not focus specifically on musculoskeletal and psychological SA. The distribution of SA duration is also skewed, which could introduce some bias into our results, though we chose the negative binomial regression method to try to account for this. This could be mitigated by using a DID approach that uses the median, though this requires a different method of analysis and additional assumptions [31].

We also controlled for variables that could cause the groups to have different levels in SA at baseline (e.g. mean company size) [5, 17]. We did not, however, have information on other potentially important confounders, such as sector (public or private) or employees’ work histories, which may influence group membership and level of SA and could thus have affected our estimates [17, 23]. Another important assumption underlying DID is that the intervention and control groups are well-defined [22], which includes the assumption that individuals cannot randomly switch group. We excluded those who switched group in 2000 or in 2005, as well as those in a different group in 2005 compared to 2000, but we included individuals who switched groups in the 4-year period between 2000 and 2005. When excluding those who changed IA status between 2000 and 2005, the results were similar to those of the original analysis. It is, however, important to follow up studies using DID with other analytical approaches, in order to understand more about the causal effects of interventions, including the IA Agreement.

We only included those in work for more than 10 months during the year (2000 or 2005), and we excluded people who switched group (intervention/control) or economic activity. These criteria could result in the exclusion of vulnerable individuals who have temporary contracts or who are struggling to find a secure and stable job, a situation that may be prevalent among our population of younger adults (aged 24–38). Applying these inclusion/exclusion criteria, though necessary to ensure proper exposure to the intervention, could limit the extent to which our findings can be applied to the general younger working population. In addition, younger individuals are less likely to experience SA compared with older adults [17] and therefore the SA levels in this study are not representative of the general working population. Finally, due to data limitations, only SA > 16 calendar days were included; this limits the generalisability of our findings to short-term SA (< 16 days).

### Implications and future research

The first goal of the IA Agreement was to reduce SA by 20% from the 2001 level [5]. This goal was not reached [4]; however, the IA Agreement may still have contributed to meaningful reductions in SA, particularly for SA duration. An example of this is the reduction in mean duration of musculoskeletal SA, which was almost 17 days in men. In addition, IA companies appear to use graded SA to keep people in contact with the workplace during illness, which aids in achieving the overarching goal of keeping people in work [4].

The variation found between outcome measure, diagnosis, gender and economic activity in this study suggests that the overall impact of the IA Agreement is considerably heterogeneous. This indicates the importance of the economic activity-specific focus in the current IA Agreement [11], and suggests the potential relevance of focusing more on gender differences. Future studies should look closer at the reasons behind the heterogeneities; for example, whether differences are due to overall implementation of specific IA-related measures, which we did not have information on in this study, or due to variance in measure implementation that may depend on, for example, company motivation or job tasks. Looking closer at economic activities such as the wholesale/retail economic activity, which showed a consistent beneficial impact of the IA Agreement in this study, may provide further insights into what aspects of the IA Agreement contribute to SA reduction. Stratifying by occupational categories would also be useful, to study differences according to job tasks. Additionally, it would be beneficial for future studies to identify gender and economic activity differences in other samples, e.g. older samples or the whole working population.

Looking beyond Norway, the results indicate that other countries considering national interventions to reduce SA may find it useful to know that such interventions could have differential impact depending on the economic activity and gender. This would allow them to tailor the intervention accordingly. Our results also indicate that there may be variations in effects dependent on which SA outcome countries are interested in reducing (prevalence versus duration). Lastly, countries considering interventions to reduce SA are recommended to implement such interventions in a way that allows for proper evaluation, e.g. by random allocation of the intervention.

## Conclusions

The impact of the IA Agreement on SA prevalence varied according to diagnosis, gender, and economic activity. The IA Agreement appeared to generally contribute towards a reduction in the duration of SA in both genders, both for musculoskeletal and psychological diagnoses. This trend was also seen in the economic activity-stratified analyses. A consistent beneficial impact of the IA Agreement was found only in the wholesale/retail economic activity. The use of graded/full SA varied, but individuals working in IA companies generally utilised graded more, compared to controls. This may indicate that graded SA is used more by IA companies to reduce full SA and keep people in contact with their workplace. Identifying what workforce and enterprise characteristics are associated with a beneficial impact of the IA Agreement, as well as which IA-related measures are most effective, will help increase the chances of achieving the IA Agreement’s goal of reducing SA.

## Supplementary Information

**Additional file 1: Supplementary Fig. 1.** Graph depicting percentage of all-cause SA in men between 1993 and 2000, in intervention and control group respectively. **Supplementary Fig. 2.** Graph depicting percentage of all-cause SA in women between 1993 and 2000, in intervention and control group respectively. **Supplementary Table 1.** Descriptive statistics for employees in the intervention group (IA) and control group (no IA) in 2000 and 2005, musculoskeletal and psychological subpopulations (those with SA > 0). **Supplementary Table 2.** Comparison of DID analyses for musculoskeletal SA prevalence and duration in original analysis to those with graded (< 100%) SA and full (100%) SA^a^. **Supplementary Table 3.** Comparison of DID analyses for psychological SA prevalence and duration in original analysis to those with graded (< 100%) SA and full (100%) SA^a^. **Supplementary Table 4.** Sickness absence prevalence in the intervention (IA) group and control (non-IA) group, by diagnosis, industry and gender. **Supplementary Table 5.** Sickness absence duration for those with SA > 0 in the intervention (IA) group and control (non-IA) group, by diagnosis, industry and gender.

## Data Availability

The data that support the findings of this study are available from Statistics Norway (FD-Trygd/Central Register of Establishments and Enterprises) and the Norwegian Labour and Welfare Administration (NAV; IA Agreement data/sickness absence diagnoses), and were collected in accordance with national guidelines. Restrictions apply to the availability of these data, which were used under license for the current study, and so are not publicly available. Contact the corresponding author for more details on data availability.

## Abbreviations

CI: confidence interval
DID: Difference-in-difference
IA (Agreement): The Norwegian Agreement for a More Inclusive Working Life
ICPC-2 code: International Classification of Primary Care code
IQR: interquartile range
NACE: Statistical Classification of Economic Activities in the European Community
NAV: Norwegian Labour and Welfare Administration
PP: percentage points
RCT: randomised controlled trial
SA: sickness absence
SD: standard deviation
SSB: Statistics Norway
