# Supplementary material for: Impact of the Norwegian Agreement for a More Inclusive Working Life on diagnosis-specific sickness absence in young adults: a difference-in-difference analysis
Source: BMC Public Health. 2022 Feb 4;22:235. doi: 10.1186/s12889-022-12636-9 (PMC8817547; doi:10.1186/s12889-022-12636-9)
Supplement: Supplementary file 1 — Additional file 1: Supplementary Fig. 1. Graph depicting percentage of all-cause SA in men between 1993 and 2000, in intervention and control group respectively. Supplementary Fig. 2. Graph depicting percentage of all-cause SA in women between 1993 and 2000, in intervention and control group respectively. Supplementary Table 1. Descriptive statistics for employees in the intervention group (IA) and control group (no IA) in 2000 and 2005, musculoskeletal and psychological subpopulations (those with SA > 0). Supplementary Table 2. Comparison of DID analyses for musculoskeletal SA prevalence and duration in original analysis to those with graded (< 100%) SA and full (100%) SAa. Supplementary Table 3. Comparison of DID analyses for psychological SA prevalence and duration in original analysis to those with graded (< 100%) SA and full (100%) SAa. Supplementary Table 4. Sickness absence prevalence in the intervention (IA) group and control (non-IA) group, by diagnosis, industry and gender. Supplementary Table 5. Sickness absence duration for those with SA > 0 in the intervention (IA) group and control (non-IA) group, by diagnosis, industry and gender. [file 12889_2022_12636_MOESM1_ESM.docx]

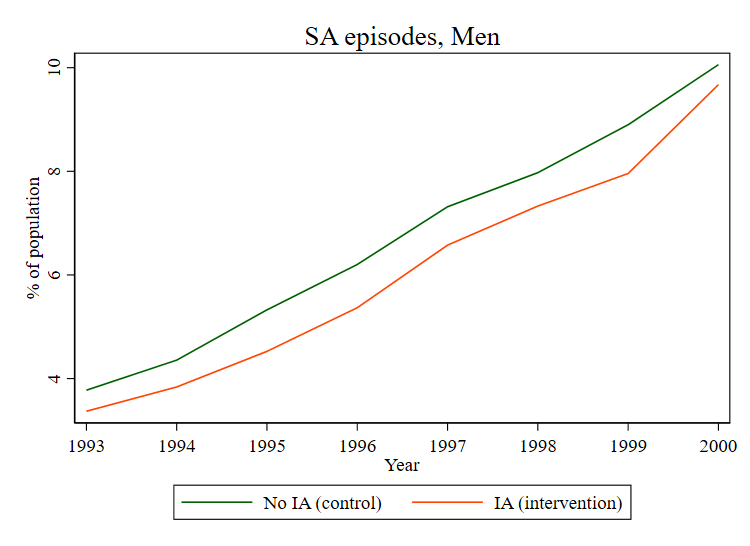
**Supplementary Figure 1.** Graph depicting percentage of all-cause SA in men between 1993-2000, in intervention and control group respectively

**Supplementary Figure 2.** Graph depicting percentage of all-cause SA in women between 1993-2000, in intervention and control group respectively


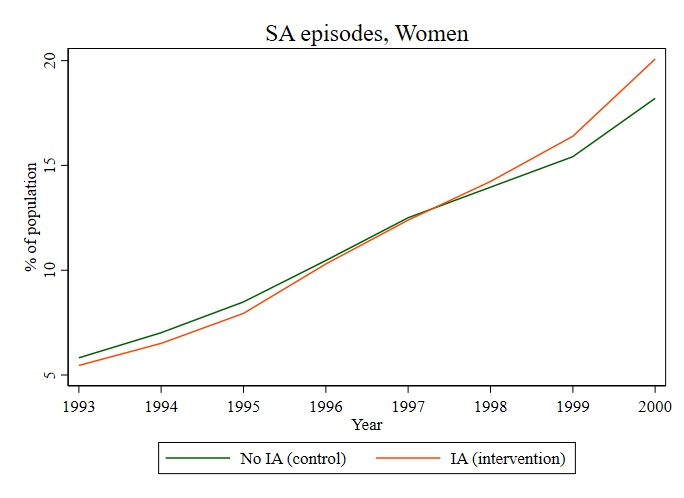


| **Supplementary Table 1.** Descriptive statistics for employees in the intervention group (IA) and control group (no IA) in 2000 and 2005, musculoskeletal and psychological subpopulations (those with SA>0) | | | | | | | | | | | | |
| --- | --- | --- | --- | --- | --- | --- | --- | --- | --- | --- | --- | --- |
|  | **Intervention** | | | | | | **Control** | | | | | |
|  | 2000 | | | 2005 | | | 2000 | | | 2005 | | |
|  | *N* | *%* | *Quartiles* | *N* | *%* | *Quartiles* | *N* | *%* | *Quartiles* | *N* | *%* | *Quartiles* |
| ***Musculoskeletal*** | N = 3,576 (22%) | | | N = 5,397 (28%) | | | N = 13,006 (78%) | | | N = 13,956 (72%) | | |
| Year of birth |  |  | 1969-1971-1973 |  |  | 1968-1970-1973 |  |  | 1969-1971-1974 |  |  | 1969-1971-1973 |
| Female | 2,543 | 71 |  | 3,383 | 63 |  | 6,380 | 49 |  | 6,061 | 43 |  |
| Company size |  |  | 44-123-417 |  |  | 42-116-434 |  |  | 8-22-79 |  |  | 9-22-68 |
| Industry |  |  |  |  |  |  |  |  |  |  |  |  |
| *Agriculture/forestry* | 11 | <1 |  | 14 | <1 |  | 317 | 2 |  | 229 | 2 |  |
| *Mining/quarrying* | 24 | 1 |  | 45 | 1 |  | 107 | 1 |  | 291 | 2 |  |
| *Manufacturing* | 587 | 16 |  | 936 | 17 |  | 2,257 | 17 |  | 2,179 | 16 |  |
| *Electricity/gas/water supply* | 13 | <1 |  | 24 | <1 |  | 43 | <1 |  | 41 | <1 |  |
| *Construction* | 167 | 5 |  | 316 | 6 |  | 1,415 | 11 |  | 1,892 | 14 |  |
| *Wholesale/retail* | 137 | 4 |  | 201 | 4 |  | 3,011 | 23 |  | 3,348 | 24 |  |
| *Service* | 82 | 2 |  | 67 | 1 |  | 813 | 6 |  | 447 | 3 |  |
| *Transport/storage* | 239 | 7 |  | 380 | 7 |  | 1,418 | 11 |  | 1,333 | 10 |  |
| *Financial/real estate* | 141 | 4 |  | 230 | 4 |  | 1,348 | 10 |  | 1,489 | 11 |  |
| *Public administration* | 234 | 7 |  | 417 | 8 |  | 279 | 2 |  | 284 | 2 |  |
| *Education* | 342 | 10 |  | 512 | 9 |  | 165 | 1 |  | 220 | 2 |  |
| *Health/social* | 1,536 | 43 |  | 2,161 | 40 |  | 1,192 | 9 |  | 1,592 | 11 |  |
| *Other* | 63 | 2 |  | 94 | 2 |  | 641 | 5 |  | 611 | 4 |  |
|  |  |  |  |  |  |  |  |  |  |  |  |  |
| ***Psychological*** | N = 1,251 (23%) | | | N = 2,892 (31%) | | | N = 4,114 (77%) | | | N = 6,343 (69%) | | |
| Year of birth |  |  | 1968-1970-1973 |  |  | 1968-1970-1973 |  |  | 1969-1971-1973 |  |  | 1969-1971-1974 |
| Female | 914 | 73 |  | 2,184 | 76 |  | 2,313 | 56 |  | 3,741 | 59 |  |
| Company size |  |  | 40-124-470 |  |  | 40-108-421 |  |  | 8-22-83 |  |  | 8-21-65 |
| Industry |  |  |  |  |  |  |  |  |  |  |  |  |
| *Agriculture/forestry* | 4 | <1 |  | 5 | <1 |  | 74 | 2 |  | 58 | 1 |  |
| *Mining/quarrying* | 2 | <1 |  | 26 | 1 |  | 36 | 1 |  | 78 | 1 |  |
| *Manufacturing* | 153 | 12 |  | 290 | 10 |  | 537 | 13 |  | 678 | 11 |  |
| *Electricity/gas/water supply* | 1 | <1 |  | 12 | <1 |  | 6 | <1 |  | 16 | <1 |  |
| *Construction* | 26 | 2 |  | 63 | 2 |  | 274 | 7 |  | 413 | 7 |  |
| *Wholesale/retail* | 44 | 4 |  | 76 | 3 |  | 900 | 22 |  | 1,563 | 25 |  |
| *Service* | 20 | 2 |  | 19 | 1 |  | 274 | 7 |  | 238 | 4 |  |
| *Transport/storage* | 76 | 6 |  | 147 | 5 |  | 424 | 10 |  | 541 | 9 |  |
| *Financial/real estate* | 52 | 4 |  | 136 | 5 |  | 611 | 15 |  | 991 | 16 |  |
| *Public administration* | 86 | 7 |  | 215 | 7 |  | 118 | 3 |  | 166 | 3 |  |
| *Education* | 159 | 13 |  | 478 | 17 |  | 92 | 2 |  | 247 | 4 |  |
| *Health/social* | 588 | 47 |  | 1,347 | 47 |  | 531 | 13 |  | 1,042 | 16 |  |
| *Other* | 40 | 3 |  | 78 | 3 |  | 237 | 6 |  | 312 | 5 |  |

| **Supplementary Table 2.** Comparison of DID analyses for musculoskeletal SA prevalence and duration in original analysis to those with graded (<100%) SA and full (100%) SA^a^ | | | | | | | | | | | | |
| --- | --- | --- | --- | --- | --- | --- | --- | --- | --- | --- | --- | --- |
|  | **Prevalence (PP)** | | | | | | **Duration (days)** | | | | | |
|  | Original Analysis | | <100% SA | | 100% SA | | Original Analysis | | <100% SA | | 100% SA | |
|  | *Marginal Change* | *95% CI* | *Marginal Change* | *95% CI* | *Marginal Change* | *95% CI* | *Marginal Change* | *95% CI* | *Marginal Change* | *95% CI* | *Marginal Change* | *95% CI* |
| Manufacturing |  |  |  |  |  |  |  |  |  |  |  |  |
| *Men* | -0.9 | -2.3, 0.4 | -0.5 | -1.1, 0.1 | -0.5 | -1.8, 0.7 | **-19.5** | **-34.1, -4.9** | -18.3 | -56.6, 20.0 | **-18.7** | **-34.8, -2.7** |
| *Women* | 1.0 | -1.3, 3.4 | 0.1 | -1.3, 1.6 | 1.0 | -1.1, 3.0 | 4.3 | -18.8, 27.4 | 19.4 | -13.7, 52.5 | -2.7 | -33.9, 28.4 |
| Construction |  |  |  |  |  |  |  |  |  |  |  |  |
| *Men* | -1.4 | -3.8, 1.1 | -0.6 | -1.8, 0.6 | -0.9 | -3.2, 1.4 | -21.5 | -44.2, 1.2 | -18.7 | -72.2, 34.7 | -19.9 | -45.3, 5.4 |
| Wholesale/retail |  |  |  |  |  |  |  |  |  |  |  |  |
| *Men* | -0.3 | -2.5, 1.8 | -0.2 | -1.4, 0.9 | -0.2 | -2.1, 1.7 | -10.1 | -44.7, 24.4 | 40.6 | -3.5, 84.7 | -22.0 | -64.2, 20.3 |
| *Women* | -2.7 | -5.8, 0.3 | -0.4 | -2.2, 1.4 | -2.5 | -5.2, 0.3 | -18.2 | -51.6, 15.1 | -5.4 | -63.5, 52.6 | -20.1 | -62.3, 22.2 |
| Transport/storage |  |  |  |  |  |  |  |  |  |  |  |  |
| *Men* | 1.2 | -1.0, 3.4 | 1.0 | -0.0, 2.1 | 0.3 | -1.8, 2.3 | -4.1 | -29.5, 21.4 | 20.1 | -42.2, 82.3 | -16.4 | -43.4, 10.7 |
| *Women* | 1.3 | -1.7, 4.3 | 2.0 | 0.0, 4.1 | -0.6 | -3.2, 2.0 | 22.7 | -4.5, 49.9 | 1.6 | -41.6, 44.8 | 24.6 | -11.2, 60.3 |
| Financial/real estate |  |  |  |  |  |  |  |  |  |  |  |  |
| *Men* | 0.5 | -1.1, 2.0 | 0.3 | -0.4, 1.0 | 0.2 | -1.2, 1.6 | -9.9 | -65.2, 45.3 | -17.7 | -125.4, 89.9 | -4.1 | -68.6, 60.4 |
| *Women* | -1.4 | -3.6, 0.9 | 0.1 | -1.2, 1.5 | -1.6 | -3.5, 0.4 | -5.0 | -34.0, 23.9 | 16.9 | -29.7, 63.5 | -10.0 | -47.2, 27.2 |
| Public administration |  |  |  |  |  |  |  |  |  |  |  |  |
| *Men* | 1.0 | -0.4, 2.5 | 0.1 | -0.5, 0.7 | 0.9 | -0.4, 2.3 | 23.8 | -10.1, 57.7 | **169.7** | **60.1, 279.3** | 5.2 | -30.4, 40.8 |
| *Women* | -1.1 | -3.1, 0.9 | -0.6 | -2.0, 0.7 | -0.5 | -2.2, 1.1 | 0.1 | -30.5, 30.7 | 14.8 | -29.6, 59.3 | -8.3 | -49.1, 32.5 |
| Education |  |  |  |  |  |  |  |  |  |  |  |  |
| *Men* | -0.1 | -1.8, 1.5 | 0.2 | -0.7, 1.1 | -0.4 | -1.9, 1.1 | -19.9 | -69.7, 29.9 | -89.0 | -185.3, 7.3 | 2.3 | -56.5, 61.2 |
| *Women* | 0.4 | -1.3, 2.0 | 1.0 | -0.1, 2.0 | -0.4 | -1.8, 0.9 | -16.5 | -44.2, 11.2 | -21.0 | -68.1, 26.1 | -18.2 | -51.7, 15.4 |
| Health/social |  |  |  |  |  |  |  |  |  |  |  |  |
| *Men* | 0.3 | -1.4, 2.1 | -0.7 | -1.6, 0.2 | 1.0 | -0.6, 2.6 | -26.4 | -59.4, 6.6 | -45.3 | -107.5, 16.9 | -26.7 | -63.9, 10.5 |
| *Women* | -0.1 | -1.1, 0.9 | 0.0 | -0.6, 0.6 | -0.1 | -1.0, 0.8 | -4.5 | -15.0, 6.0 | -8.9 | -27.9, 10.1 | -5.8 | -18.5, 6.9 |
| ^a^ Analyses adjusted for mean company size. **Bold font** indicates estimates are statistically significant at the 5% level.  *PP = percentage points, CI = confidence interval, SA = sickness absence* | | | | | | | | | | | | |

| **Supplementary Table 3.** Comparison of DID analyses for psychological SA prevalence and duration in original analysis to those with graded (<100%) SA and full (100%) SA^a^ | | | | | | | | | | | | |
| --- | --- | --- | --- | --- | --- | --- | --- | --- | --- | --- | --- | --- |
|  | **Prevalence (PP)** | | | | | | **Duration (days)** | | | | | |
|  | Original Analysis | | <100% SA | | 100% SA | | Original Analysis | | <100% SA | | 100% SA | |
|  | *Marginal Change* | *95% CI* | *Marginal Change* | *95% CI* | *Marginal Change* | *95% CI* | *Marginal Change* | *95% CI* | *Marginal Change* | *95% CI* | *Marginal Change* | *95% CI* |
| Manufacturing |  |  |  |  |  |  |  |  |  |  |  |  |
| *Men* | -0.4 | -1.1, 0.3 | 0.1 | -0.1, 0.4 | -0.5 | -1.2, 0.1 | -8.5 | -42.6, 25.6 | **59.8** | **2.3, 117.2** | -13.8 | -51.7, 24.0 |
| *Women* | -0.4 | -1.8, 1.0 | -0.6 | -1.4, 0.2 | 0.1 | -1.0, 1.3 | 18.8 | -25.0, 62.7 | -25.3 | -96.8, 46.2 | 31.2 | -22.9, 85.3 |
| Construction |  |  |  |  |  |  |  |  |  |  |  |  |
| *Men* | 0.0 | -1.0, 1.1 | -0.2 | -0.7, 0.3 | 0.2 | -0.7, 1.1 | -32.2 | -93.7, 29.3 | -45.3 | -116.7, 26.1 | -27.9 | -102.3, 46.5 |
| Wholesale/retail |  |  |  |  |  |  |  |  |  |  |  |  |
| *Men* | **-1.6** | **-3.1, -0.1** | -0.5 | -1.2, 0.3 | -1.2 | -2.5, 0.1 | -11.0 | -65.3, 43.4 | 20.8 | -93.6, 135.2 | -24.7 | -86.0, 36.6 |
| *Women* | -0.9 | -2.6, 0.7 | -0.4 | -1.3, 0.5 | -0.5 | -2.0, 0.9 | -31.4 | -94.1, 31.4 | 12.7 | -58.0, 83.5 | -43.1 | -119.5, 33.3 |
| Transport/storage |  |  |  |  |  |  |  |  |  |  |  |  |
| *Men* | -0.5 | -1.8, 0.8 | 0.1 | -0.5, 0.7 | -0.6 | -1.7, 0.5 | **45.7** | **4.6, 86.7** | 95.4 | -7.3, 198.2 | 24.8 | -18.2, 67.7 |
| *Women* | 0.9 | -1.1, 2.9 | 0.3 | -0.9, 1.4 | 0.7 | -1.1, 2.5 | 0.3 | -50.3, 50.8 | 34.7 | -36.3, 105.6 | -12.0 | -74.8, 50.8 |
| Financial/real estate |  |  |  |  |  |  |  |  |  |  |  |  |
| *Men* | 0.1 | -0.9, 1.1 | -0.1 | -0.7, 0.4 | 0.3 | -0.6, 1.1 | **55.6** | **4.8, 106.3** | 29.7 | -62.0, 121.5 | **62.3** | **1.7, 122.9** |
| *Women* | 0.2 | -1.4, 1.7 | 0.2 | -0.7, 1.1 | 0.0 | -1.3, 1.4 | -30.5 | -81.9, 20.8 | -46.5 | -133.6, 40.6 | -29.2 | -92.7, 34.4 |
| Public administration |  |  |  |  |  |  |  |  |  |  |  |  |
| *Men* | 0.8 | -0.1, 1.7 | 0.0 | -0.5, 0.6 | 0.7 | -0.1, 1.5 | -9.0 | -75.6, 57.6 | 88.2 | -16.5, 192.9 | -43.2 | -131.5, 45.1 |
| *Women* | -0.5 | -2.0, 0.9 | -0.2 | -1.1, 0.8 | -0.4 | -1.5, 0.7 | 1.7 | -47.8, 51.2 | -26.1 | -89.1, 36.9 | 12.1 | -60.6, 84.7 |
| Education |  |  |  |  |  |  |  |  |  |  |  |  |
| *Men* | 0.2 | -1.2, 1.6 | -0.1 | -0.8, 0.6 | 0.3 | -0.9, 1.5 | -38.6 | -102.6, 25.4 | **-151.3** | **-293.2, 9.3** | -1.9 | -74.4, 70.5 |
| *Women* | -0.1 | -1.5, 1.3 | 0.2 | -0.7, 1.1 | -0.4 | -1.5, 0.7 | 20.9 | -18.2, 60.0 | 40.3 | -31.0, 111.6 | -1.9 | -50.8, 47.1 |
| Health/social |  |  |  |  |  |  |  |  |  |  |  |  |
| *Men* | 1.2 | -0.2, 2.5 | 0.5 | -0.3, 1.2 | 0.7 | -0.5, 1.9 | -22.3 | -70.8, 26.2 | -10.5 | -101.9, 80.9 | -22.0 | -79.4, 35.4 |
| *Women* | 0.1 | -0.7, 0.8 | -0.2 | -0.6, 0.3 | 0.3 | -0.4, 0.9 | -7.1 | -23.9, 9.7 | -12.8 | -43.6, 17.9 | -6.6 | -27.0, 13.7 |
| ^a^ Analyses adjusted for mean company size. **Bold font** indicates estimates are statistically significant at the 5% level.  *PP = percentage points, CI = confidence interval, SA = sickness absence* | | | | | | | | | | | | |

| **Supplementary Table 4.** Sickness absence prevalence in the intervention (IA) group and control (non-IA) group, by diagnosis, industry and gender | | | | | | | | |
| --- | --- | --- | --- | --- | --- | --- | --- | --- |
|  | **2000** | | | | **2005** | | | |
|  | Intervention | | Control | | Intervention | | Control | |
| **Musculoskeletal (code L)** | N | % | N | % | N | % | N | % |
| Agriculture/forestry |  |  |  |  |  |  |  |  |
| *Men* | 4 | 7 | 204 | 7 | 10 | 11 | 184 | 9 |
| *Women* | 7 | 19 | 113 | 9 | 4 | 10 | 45 | 7 |
| Mining/quarrying |  |  |  |  |  |  |  |  |
| *Men* | 8 | 3 | 88 | 7 | 26 | 3 | 249 | 7 |
| *Women* | 16 | 7 | 19 | 6 | 19 | 4 | 42 | 7 |
| Manufacturing |  |  |  |  |  |  |  |  |
| *Men* | 402 | 11 | 1,538 | 11 | 691 | 8 | 1,637 | 8 |
| *Women* | 185 | 13 | 719 | 13 | 245 | 10 | 542 | 9 |
| Electricity/gas/water supply |  |  |  |  |  |  |  |  |
| *Men* | 8 | 6 | 31 | 6 | 18 | 5 | 31 | 4 |
| *Women* | 5 | 8 | 12 | 7 | 6 | 5 | 10 | 4 |
| Construction |  |  |  |  |  |  |  |  |
| *Men* | 147 | 14 | 1,327 | 12 | 295 | 11 | 1,826 | 11 |
| *Women* | 20 | 10 | 88 | 10 | 21 | 8 | 66 | 6 |
| Wholesale/retail |  |  |  |  |  |  |  |  |
| *Men* | 61 | 8 | 1,298 | 7 | 107 | 6 | 1,570 | 6 |
| *Women* | 76 | 12 | 1,713 | 10 | 94 | 9 | 1,778 | 9 |
| Service |  |  |  |  |  |  |  |  |
| *Men* | 10 | 6 | 213 | 6 | 12 | 7 | 133 | 7 |
| *Women* | 72 | 14 | 600 | 10 | 55 | 13 | 314 | 10 |
| Transport/storage |  |  |  |  |  |  |  |  |
| *Men* | 112 | 9 | 945 | 10 | 214 | 10 | 996 | 9 |
| *Women* | 127 | 13 | 473 | 7 | 166 | 13 | 337 | 9 |
| Financial/real estate |  |  |  |  |  |  |  |  |
| *Men* | 28 | 4 | 520 | 4 | 75 | 3 | 653 | 3 |
| *Women* | 113 | 10 | 828 | 7 | 155 | 8 | 836 |  |
| Public administration |  |  |  |  |  |  |  |  |
| *Men* | 56 | 4 | 123 | 3 | 166 | 4 | 123 | 3 |
| *Women* | 178 | 8 | 156 | 7 | 251 | 7 | 161 | 7 |
| Education |  |  |  |  |  |  |  |  |
| *Men* | 56 | 3 | 31 | 3 | 116 | 3 | 59 | 3 |
| *Women* | 286 | 7 | 134 | 7 | 396 | 5 | 161 | 5 |
| Health/social |  |  |  |  |  |  |  |  |
| *Men* | 125 | 5 | 115 | 6 | 249 | 6 | 201 | 7 |
| *Women* | 1,411 | 11 | 1,077 | 11 | 1,912 | 9 | 1,391 | 10 |
| Other |  |  |  |  |  |  |  |  |
| *Men* | 16 | 4 | 193 | 5 | 35 | 6 | 233 | 6 |
| *Women* | 47 | 7 | 448 | 10 | 59 | 8 | 378 | 9 |
|  |  |  |  |  |  |  |  |  |
| **Psychological (code P)** |  |  |  |  |  |  |  |  |
| Agriculture/forestry |  |  |  |  |  |  |  |  |
| *Men* | 4 | 7 | 42 | 2 | 2 | 2 | 35 | 2 |
| *Women* | 0 | 0 | 32 | 3 | 3 | 8 | 23 | 4 |
| Mining/quarrying |  |  |  |  |  |  |  |  |
| *Men* | 1 | <1 | 29 | 2 | 10 | 1 | 54 | 1 |
| *Women* | 1 | <1 | 7 | 2 | 16 | 4 | 24 | 4 |
| Manufacturing |  |  |  |  |  |  |  |  |
| *Men* | 103 | 3 | 341 | 2 | 185 | 2 | 396 | 2 |
| *Women* | 50 | 3 | 196 | 4 | 105 | 4 | 282 | 5 |
| Electricity/gas/water supply |  |  |  |  |  |  |  |  |
| *Men* | 1 | 1 | 4 | 1 | 7 | 2 | 8 | 1 |
| *Women* | 0 | 0 | 2 | 1 | 5 | 4 | 8 | 3 |
| Construction |  |  |  |  |  |  |  |  |
| *Men* | 22 | 2 | 251 | 2 | 52 | 2 | 370 | 2 |
| *Women* | 4 | 2 | 23 | 3 | 11 | 4 | 43 | 4 |
| Wholesale/retail |  |  |  |  |  |  |  |  |
| *Men* | 28 | 3 | 342 | 2 | 40 | 2 | 601 | 2 |
| *Women* | 16 | 3 | 558 | 3 | 36 | 3 | 962 | 5 |
| Service |  |  |  |  |  |  |  |  |
| *Men* | 3 | 2 | 77 | 2 | 0 | 0 | 76 | 4 |
| *Women* | 17 | 3 | 197 | 3 | 19 | 5 | 162 | 5 |
| Transport/storage |  |  |  |  |  |  |  |  |
| *Men* | 35 | 3 | 261 | 3 | 62 | 3 | 346 | 3 |
| *Women* | 41 | 4 | 163 | 3 | 85 | 7 | 195 | 5 |
| Financial/real estate |  |  |  |  |  |  |  |  |
| *Men* | 11 | 1 | 236 | 2 | 39 | 2 | 412 | 2 |
| *Women* | 41 | 4 | 375 | 3 | 97 | 5 | 579 | 4 |
| Public administration |  |  |  |  |  |  |  |  |
| *Men* | 19 | 1 | 48 | 1 | 63 | 2 | 55 | 1 |
| *Women* | 67 | 3 | 70 | 3 | 152 | 4 | 111 | 5 |
| Education |  |  |  |  |  |  |  |  |
| *Men* | 31 | 2 | 23 | 2 | 76 | 2 | 47 | 2 |
| *Women* | 128 | 3 | 69 | 4 | 402 | 6 | 200 | 6 |
| Health/social |  |  |  |  |  |  |  |  |
| *Men* | 69 | 3 | 73 | 4 | 150 | 3 | 111 | 4 |
| *Women* | 519 | 4 | 458 | 5 | 1,197 | 6 | 931 | 7 |
| Other |  |  |  |  |  |  |  |  |
| *Men* | 10 | 2 | 74 | 2 | 22 | 4 | 91 | 3 |
| *Women* | 30 | 4 | 163 | 4 | 56 | 7 | 221 | 5 |

| **Supplementary Table 5.** Sickness absence duration for those with SA>0 in the intervention (IA) group and control (non-IA) group, by diagnosis, industry and gender | | | | | | | | |
| --- | --- | --- | --- | --- | --- | --- | --- | --- |
|  | **2000** | | | | **2005** | | | |
|  | Intervention | | Control | | Intervention | | Control | |
| **Musculoskeletal (code L)** | Mean | SD | Mean | SD | Mean | SD | Mean | SD |
| Agriculture/forestry |  |  |  |  |  |  |  |  |
| *Men* | 149 | 139 | 104 | 103 | 125 | 86 | 132 | 124 |
| *Women* | 101 | 65 | 120 | 104 | 72 | 79 | 137 | 118 |
| Mining/quarrying |  |  |  |  |  |  |  |  |
| *Men* | 41 | 26 | 84 | 82 | 84 | 85 | 88 | 91 |
| *Women* | 78 | 73 | 109 | 76 | 110 | 102 | 115 | 118 |
| Manufacturing |  |  |  |  |  |  |  |  |
| *Men* | 102 | 103 | 97 | 98 | 90 | 99 | 105 | 109 |
| *Women* | 107 | 87 | 108 | 97 | 130 | 118 | 127 | 121 |
| Electricity/gas/water supply |  |  |  |  |  |  |  |  |
| *Men* | 51 | 34 | 94 | 90 | 60 | 61 | 66 | 62 |
| *Women* | 102 | 83 | 154 | 142 | 116 | 124 | 127 | 105 |
| Construction |  |  |  |  |  |  |  |  |
| *Men* | 109 | 101 | 109 | 104 | 110 | 110 | 130 | 119 |
| *Women* | 120 | 102 | 105 | 91 | 132 | 108 | 163 | 137 |
| Wholesale/retail |  |  |  |  |  |  |  |  |
| *Men* | 100 | 105 | 104 | 103 | 103 | 109 | 116 | 112 |
| *Women* | 105 | 105 | 110 | 96 | 111 | 107 | 135 | 122 |
| Service |  |  |  |  |  |  |  |  |
| *Men* | 93 | 92 | 107 | 104 | 75 | 96 | 129 | 125 |
| *Women* | 118 | 98 | 119 | 101 | 172 | 131 | 154 | 129 |
| Transport/storage |  |  |  |  |  |  |  |  |
| *Men* | 91 | 94 | 102 | 102 | 102 | 108 | 117 | 113 |
| *Women* | 81 | 78 | 97 | 89 | 124 | 123 | 117 | 114 |
| Financial/real estate |  |  |  |  |  |  |  |  |
| *Men* | 120 | 130 | 106 | 105 | 104 | 102 | 108 | 108 |
| *Women* | 117 | 106 | 105 | 99 | 123 | 116 | 120 | 113 |
| Public administration |  |  |  |  |  |  |  |  |
| *Men* | 58 | 71 | 87 | 91 | 78 | 82 | 75 | 86 |
| *Women* | 92 | 98 | 91 | 83 | 114 | 114 | 114 | 120 |
| Education |  |  |  |  |  |  |  |  |
| *Men* | 80 | 84 | 85 | 95 | 70 | 86 | 93 | 95 |
| *Women* | 109 | 97 | 98 | 82 | 113 | 114 | 118 | 117 |
| Health/social |  |  |  |  |  |  |  |  |
| *Men* | 106 | 111 | 96 | 99 | 90 | 96 | 106 | 110 |
| *Women* | 94 | 85 | 105 | 94 | 107 | 109 | 123 | 117 |
| Other |  |  |  |  |  |  |  |  |
| *Men* | 136 | 131 | 93 | 91 | 89 | 93 | 113 | 108 |
| *Women* | 101 | 78 | 125 | 102 | 138 | 131 | 146 | 127 |
|  |  |  |  |  |  |  |  |  |
| **Psychological (code P)** |  |  |  |  |  |  |  |  |
| Agriculture/forestry |  |  |  |  |  |  |  |  |
| *Men* | 106 | 122 | 109 | 114 | 23 | 4 | 122 | 113 |
| *Women* | - | - | 122 | 121 | 134 | 142 | 196 | 132 |
| Mining/quarrying |  |  |  |  |  |  |  |  |
| *Men* | 25 | - | 99 | 101 | 109 | 131 | 90 | 96 |
| *Women* | 361 | - | 39 | 22 | 168 | 111 | 95 | 95 |
| Manufacturing |  |  |  |  |  |  |  |  |
| *Men* | 130 | 126 | 139 | 120 | 109 | 113 | 125 | 127 |
| *Women* | 112 | 105 | 136 | 120 | 146 | 128 | 151 | 129 |
| Electricity/gas/water supply |  |  |  |  |  |  |  |  |
| *Men* | 40 | - | 64 | 45 | 103 | 94 | 169 | 143 |
| *Women* | - | - | 174 | 223 | 235 | 133 | 184 | 139 |
| Construction |  |  |  |  |  |  |  |  |
| *Men* | 122 | 120 | 117 | 107 | 96 | 106 | 124 | 112 |
| *Women* | 79 | 54 | 152 | 138 | 111 | 129 | 157 | 128 |
| Wholesale/retail |  |  |  |  |  |  |  |  |
| *Men* | 100 | 118 | 137 | 119 | 89 | 92 | 136 | 122 |
| *Women* | 114 | 103 | 131 | 118 | 101 | 108 | 150 | 128 |
| Service |  |  |  |  |  |  |  |  |
| *Men* | 88 | 64 | 128 | 110 | - | - | 152 | 129 |
| *Women* | 100 | 105 | 152 | 129 | 142 | 117 | 157 | 132 |
| Transport/storage |  |  |  |  |  |  |  |  |
| *Men* | 79 | 72 | 132 | 120 | 114 | 111 | 122 | 116 |
| *Women* | 118 | 115 | 120 | 113 | 126 | 126 | 128 | 123 |
| Financial/real estate |  |  |  |  |  |  |  |  |
| *Men* | 54 | 44 | 116 | 115 | 127 | 129 | 132 | 123 |
| *Women* | 150 | 140 | 128 | 120 | 126 | 119 | 137 | 122 |
| Public administration |  |  |  |  |  |  |  |  |
| *Men* | 107 | 106 | 82 | 91 | 102 | 108 | 98 | 110 |
| *Women* | 93 | 108 | 106 | 109 | 129 | 119 | 149 | 126 |
| Education |  |  |  |  |  |  |  |  |
| *Men* | 101 | 104 | 100 | 80 | 97 | 86 | 135 | 130 |
| *Women* | 119 | 114 | 151 | 118 | 131 | 114 | 143 | 120 |
| Health/social |  |  |  |  |  |  |  |  |
| *Men* | 130 | 113 | 139 | 121 | 109 | 110 | 142 | 128 |
| *Women* | 106 | 105 | 111 | 108 | 117 | 116 | 129 | 120 |
| Other |  |  |  |  |  |  |  |  |
| *Men* | 128 | 128 | 127 | 116 | 167 | 132 | 128 | 120 |
| *Women* | 142 | 115 | 139 | 114 | 180 | 129 | 158 | 133 |

*SD = standard deviation*
